# Point-of-Care PCR Assays for COVID-19 Detection

**DOI:** 10.3390/bios11050141

**Published:** 2021-05-01

**Authors:** Niharika Gupta, Shine Augustine, Tarun Narayan, Alan O’Riordan, Asmita Das, D. Kumar, John H. T. Luong, Bansi D. Malhotra

**Affiliations:** 1 Department of Biotechnology, Delhi Technological University, Shahbad Daulatpur, Delhi 110042, India; niharika.gupta990@gmail.com (N.G.); shine2089@gmail.com (S.A.); asmita1710@gmail.com (A.D.); 2 Nanotechnology Group, Tyndall National Institute, University College Cork, T12 K8AF Cork, Ireland; tarun.narayan@tyndall.ie (T.N.); alan.oriordan@tyndall.ie (A.O.); 3 Department of Applied Chemistry, Delhi Technological University, Shahbad Daulatpur, New Delhi 110042, India; dkumar@dce.ac.in; 4 School of Chemistry, University College Cork, T12 K8AF Cork, Ireland

**Keywords:** polymerase chain reaction, COVID-19, electrochemical, digital PCR, point-of-care

## Abstract

Molecular diagnostics has been the front runner in the world’s response to the COVID-19 pandemic. Particularly, reverse transcriptase-polymerase chain reaction (RT-PCR) and the quantitative variant (qRT-PCR) have been the gold standard for COVID-19 diagnosis. However, faster antigen tests and other point-of-care (POC) devices have also played a significant role in containing the spread of SARS-CoV-2 by facilitating mass screening and delivering results in less time. Thus, despite the higher sensitivity and specificity of the RT-PCR assays, the impact of POC tests cannot be ignored. As a consequence, there has been an increased interest in the development of miniaturized, high-throughput, and automated PCR systems, many of which can be used at point-of-care. This review summarizes the recent advances in the development of miniaturized PCR systems with an emphasis on COVID-19 detection. The distinct features of digital PCR and electrochemical PCR are detailed along with the challenges. The potential of CRISPR/Cas technology for POC diagnostics is also highlighted. Commercial RT–PCR POC systems approved by various agencies for COVID-19 detection are discussed.

## 1. Introduction

The coronavirus disease 2019 (COVID-19) outbreak crisis has changed the shape of our world since its first report in December 2019. While some countries seem to be recovering from the crisis and are reporting fewer cases, others are still witnessing an increasing number of cases [1]. Clinical diagnosis has been the forerunner in controlling the COVID-19 pandemic. Molecular nucleic acid amplification tests (NAATs) were the first to be developed for detecting SARS-CoV-2 RNA in patient samples. Particularly, reverse transcriptase-polymerase chain reaction (RT-PCR) and its quantitative variant (qRT-PCR) have been the keystone for diagnosis of SARS-CoV-2 with the capacity to detect target nucleic acids (<100 copies/mL) with remarkable sensitivity [2]. However, the analysis proved time-intensive, requiring up to a few hours, and could only be performed in a centralized laboratory. The high false-negative rates with some RT-PCR assays also raised concern. Thus, attention shifted to faster, cheaper, and equally sensitive (if not more) point-of-care (POC) biosensing devices that could be deployed for mass screening.

Therein began a major shift in the clinical diagnostic industry, with point-of-care testing (POCT) becoming the focus of attention almost overnight. Lateral flow assays (LFAs), chemiluminescence, and nanoparticle-based colorimetric detection were developed for detecting SARS-CoV-2-related antigens and antibodies produced in response to its infection [3,4,5,6,7,8]. Faster, miniaturized isothermal amplification tests emerged that could detect the virus within a few minutes and with sensitivity at par with RT-PCR assays [5,9,10]. Although different types of POCT devices have been authorized in various countries for emergency use, many novel biosensing strategies and designs still seek validation and are currently subject to academic inquiry.

These devices have shorter response times and have cost-effectively enabled population-wide mass screening. However, evidence suggests that the analytic performance (sensitivity, specificity, positive and negative predictive values, etc.) of current antigen diagnostic tests is not at par with that of RT-PCR and other NAATs [11]. Thus, while rapid antigen tests and other POCT are being widely used for COVID-19 screening, it is still uncertain whether such tests will be regularized and used in routine diagnostic procedures. In attempt to synergize the sensitivity of NAATs and the ease of use of POCT assays, miniaturized NAAT-based POCT devices and assays were devised for faster screening and diagnosis of COVID-19. One of the first such devices was the Abbott ID Now, which integrates isothermal amplification with colorimetric detection to yield results within 5 min. However, questions were soon raised about its utility as a singular diagnostic test due to its low positive predictive value (PPA) and high false-negative rates, especially in samples with low viral load [10]. More rapid devices based on isothermal amplification with improved performance were devised. Thus, although the integration of isothermal amplification in POC devices has gained some success, they are not as successful as RT-PCR for COVID-19 detection. In general, the high temperature requirements of RT-PCR prevent non-specific amplification, which is more common in isothermal amplification techniques. Conversely, these temperature requirements somewhat complicate the development of PCR-based rapid devices.

Nonetheless, efforts have been directed toward miniaturizing PCR to make it an automated, high-throughput device that can be applied at point-of-use. In this review, we summarize studies related to the development of miniaturized, high-throughput PCR biosensors for COVID-19 detection. The distinct features, limitations, and advantages of various types of PCR biosensors and chips are discussed. The advantages and limitations of PCR chips over biosensors based on other amplification assays are listed. The potential of biosensing formats to be integrated with RT-PCR is explored, along with the path-breaking integration of CRISPR/Cas technology with amplification assays toward the development of faster, miniaturized devices and chips.

## 2. RT-PCR: The Gold Standard

RT-PCR is the first molecular diagnostic test to be employed for detecting SARS-CoV-2 RNA in patient samples and is currently considered the gold standard for COVID-19 diagnosis. Different RT-PCR assays have been designed for detecting SARS-CoV-2 virus RNA in different body fluids, such as nasopharyngeal swabs, lower respiratory tract fluid, sputum, saliva, etc. [12,13,14]. However, RT-PCR is prone to false-negative results that reduce the overall sensitivity of the diagnosis. This may be because of various reasons such as low viral load in the pharyngeal, nasal, and sputum samples; storage and transport of samples; and improper handling [15,16]. Moreover, any mismatches between the primers and probe–target regions compromise the assay performance, leading to false-negative results [15,17]. Another major challenge faced by RT-PCR is that it can yield false-positive results by amplifying RNA from dead, noninfectious viruses as well [18]. Thus, recovered patients that no longer hold the threat of transmitting the disease may be positive per RT-PCR tests.

The current challenges of the qRT-PCR method include the use of fluorescent label binding to the source signal produced by the amplified DNA, which not only increases the cost of the instrument, but also the complexities. This technology is less appealing to developing nations or remote locations with limited resources. Commercial RT-PCR kits have not been subject to rigorous quality control. Personnel skills and good laboratory practice play an important role in Biosafety Level 3. Optimum sample types and timing for peak viral load remain to be fully investigated as sputum or nasal swabs are the most accurate sample for diagnosis of COVID-19, but not throat swabs.

**Figure 1:**
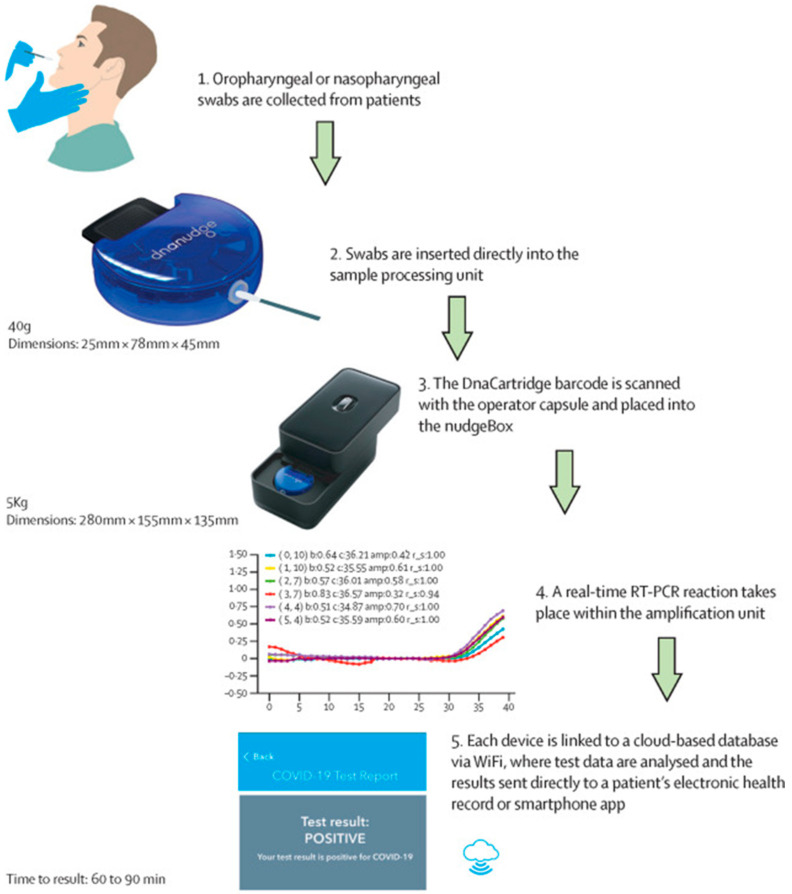
Schematic diagram depicting the various steps performed by the CovidNudge assay for automated detection of SARS-CoV-2 RNA (Reprinted with permission from Ref. [20]).

Despite these limitations, RT-PCR remains the gold standard for confirming the diagnosis of COVID-19. There have been multiple attempts to develop portable PCR systems since the inception of the pandemic. Lab-in-tube systems incorporating lysis, reverse transcription, amplification, and detection in a single tube within 36 min were demonstrated in May 2020 [19]. A lab-on-chip device, CovidNudge, can be used to perform sample processing and real-time RT-PCR outside of a laboratory setting [20] (Figure 1). The chip consists of detection arrays for seven SARS-CoV-2 genes and one host gene as a sample adequacy control. This device detects the virus in 90 min and reduces the collection-to-result turnaround time significantly by eliminating the requirement of sample transport from the site of collection to a centralized lab. The sensitivity of this POC test (94%) is comparable to that of lab-based tests in clinical settings. As of September 2020, over 5 M CovidNudge kits had been deployed in the U.K. for COVID-19 testing. Of note is a portable RT-PCR workstation for COVID-19 detection in under-served and remote areas [21]. This workstation is a chip-based, battery-operated qRT-PCR system with the capability of network data transfer and automated reporting. Almost 3.8% (2.7 million) of the total tests conducted in India were performed on these workstations (as of September 2020). The average cost of an RT-PCR varies in different parts of the world. In India, for example, the cost of a conventional RT-PCR test currently varies from INR 400 (~USD 5.30) to INR 950 (~USD 12.6), and POC rapid antigen tests are free. While the CovidNudge test (Table 1) deployed in the U.K. costs around GBP 10 (per test) (equivalent to ~USD 13.80), which is almost 10 times cheaper than the average cost (~GBP 100) of a conventional RT-PCR test in the country.

There have been several other innovations related to the fabrication of PCR chips and biosensors for COVID-19 detection. The following sections cover some of these studies and discuss the potential and challenges faced by such devices in emerging as viable commercial products.

## 3. RT-PCR Biosensors

### 3.1. Digital RT-PCR

**Figure 2:**
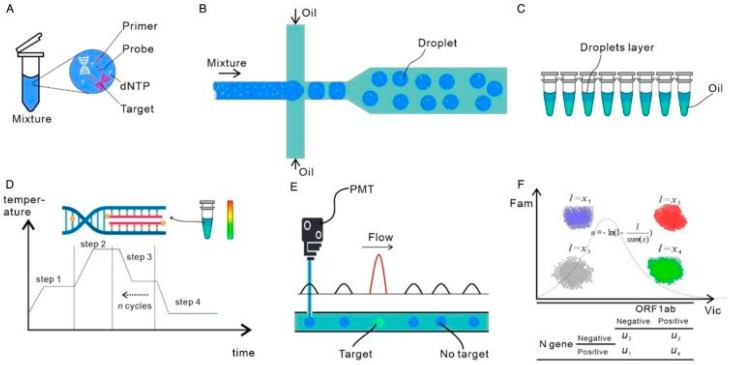
Schematic depicting workflow of a ddPCR system: (**A**) preparation for amplification, (**B**) generation of water-in-oil droplets using a microfluidic flow system, (**C**) collection of the droplets in PCR tubes, (**D**) PCR amplification, (**E**) analysis of fluorescence in the droplets after amplification, and (**F**) fitting to Poisson distribution to determine the absolute copy numbers of the target molecules (Reprinted from Ref. [28]).

The concept of digital PCR (dPCR) was pioneered by Vogelstein and Kinzler in 1999 [24]. The principle of dPCR is to partition the reaction mixture into many sub-reactions before amplification; the original numbers are determined by counting the partition showing negative and positive reactions [25] (Figure 2). It does not require a standard curve or reference genes and is more resistant to interference factors such as specific template amplification inhibitors [26,27]. The quantification results are analyzed from Poisson’s distribution and can achieve an accurate estimation of low concentrations of nucleic acid samples [26]. Therefore, a method like dPCR offers high sensitivity, higher precision, and resistance to inhibitors, which are required for an accurate SARS-CoV-2 diagnosis. The dPCR method can be classified into three types based on liquid separation: droplet-based (ddPCR), chip-based (cdPCR), and microfluidic digital PCR (mdPCR). The primary difference between these three types of digital PCR is the design of the sample partitioning system in the detection platform: ddPCR combines several millions partitioning of the PCR test into individual droplets in a water-in-oil emulsion [26,28], whereas cdPCR uses an active partitioning approach. It has two chip halves with two arrays of microwells. The chambers are aligned so that the opposite halves form continuous channels [28,29]. In mdPCR, microfluidic chambers are used to split the samples. These chambers are fluidically designed such that each sample can be partitioned into tens of thousands of wells [30].

dPCR can be used for the quantification of a low viral load, monitoring of the virus in the environment, evaluation of anti-SARS-CoV-2 drugs [28], and the detection of viral mutations [31]. Many types of clinical samples can be used for COVID-19 testing using dPCR, including blood, urine, sputum, stool, nasal swabs, and throat swabs. Studies have compared RT-PCR with RT-dPCR for the presence of SARS-CoV-2 in pharyngeal swab samples and found RT-dPCR to be more sensitive and accurate than RT-PCR [32,33]. Lu and group showed that RT-dPCR has a detection limit ten-fold lower than that of RT-PCR [34]. They compared the RT-dPCR and RT-PCR of 36 COVID-19 patients with 108 specimens, including blood, pharyngeal swab, and stool, in which four pharyngeal samples yielding negative results in RT-PCR were positive per RT-dPCR. Another study demonstrated that suspected patients who tested negative by RT-PCR were found to be positive by ddPCR [35]. The results of ddPCR were validated by the serological testing of anti-COVID-19 antibodies in the samples. The ddPCR can yield better and more precise quantitation of viral loads of SARS-CoV-2 [36,37,38]. However, most of the reported ddPCR procedures included an RNA extraction and purification step, which can lead to potential amplification errors [38]. Moreover, direct quantification by ddPCR targeting the envelope (E) gene [39], ORF1ab gene [40], and nucleocapsid (N) [41] region have also been reported. The viral load can be quantified in throat swabs, sputum, nasal swabs, blood, and urine [37]. Droplet-based dPCR was also used to detect SARS-CoV-2 RNA in airborne aerosols [42], in which the viral load in the toilets used by some medical personnel and patients was found to be high. This study indicated the significance of sanitization and room ventilation for limiting COVID-19 spread. The primary advantage of dPCR is its good sensitivity and high-throughput analysis, which has been the key requirement for COVID-19 detection. Currently, there are three commercial dPCR tests authorized for emergency use by the USFDA (Table 1).

However, a few challenges require the utmost attention before dPCR can be used in routine diagnostics. Particularly, much like conventional PCR tests, dPCR also requires expensive instruments, reagents, and professional experts to operate the system. The fabrication of the dPCR chips requires complex steps, making it a costly operation. Moreover, much like other POC tests, strict standards and guidelines need to be followed to assure the quality of results obtained from dPCR systems.

### 3.2. Electrochemical PCR: Unexplored Potential

The integration of electrochemistry with RT-PCR aims to provide a rapid, miniaturized, hand-held instrument. Electrochemical biosensors work by modification of a working electrode with a biomolecule that interacts with a specific target analyte present in an aqueous electrolyte and generates an electrical signal corresponding to its concentration. In the case of an electrochemical PCR, there is an electroactive species whose oxidation or reduction signal is correlated to the amount of PCR amplified product. A more challenging approach is the use of nanomaterials to tag the DNA primers used in the PCR amplification step, such as gold nanoparticles (AuNPs) or semiconductor quantum dots (QDs). The labeled amplified products are then further quantified via the generation of electrochemical signals.

Electrochemical systems offer the benefits of being seamlessly implemented into compact and intelligent systems, enabling high versatility and real-time detection. Moreover, electrochemically active labels (such as metal-complex, organic molecules, etc.) are more durable than fluorescent dyes (Cy5, FAM, etc.) and are a notable factor toward the commercial applications of electrochemical-RT-PCR (EPCR). The power and sample volume requirements are lower for electrochemical biosensors compared with RT-PCR. Despite the considerable interest, electrochemical biosensors have garnered in the context of COVID-19 detection, the clinical industry appears reluctant to adopt this technology for practical and commercial use.

The pre-COVID era witnessed the emergence of PCR-free electrochemical assays for detecting different nucleic acid targets, including microRNA, viral RNA and DNA, and cancer-related genes [43,44,45]. Perhaps the research community has been confident that electrochemical assays can compete with the existing PCR technology in terms of sensitivity and turnaround times and eliminate the use of costly reagents and dyes [46]. There have been some studies on PCR-integrated electrochemical biosensors in the last 5 years. Some of the recent studies have demonstrated innovative PCR-free electrochemical sensors for SARS-CoV-2 RNA detection with remarkable detection limits [47,48]; however, none has yet achieved a commercial or authorized status.

**Figure 3:**
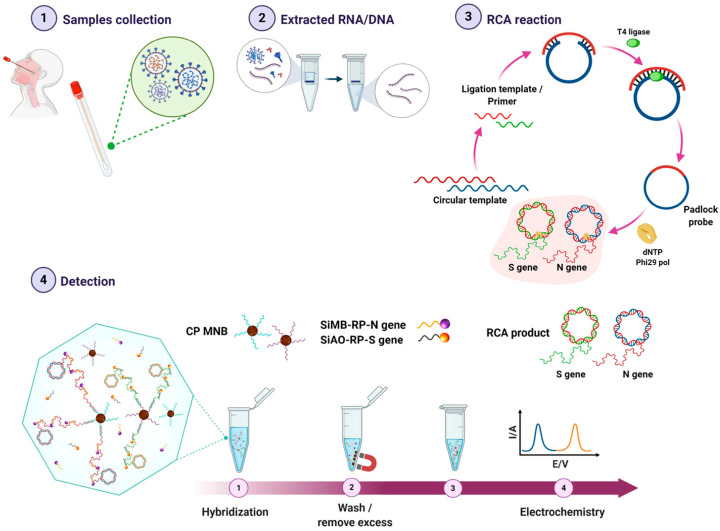
Workflow of the RCA-based electrochemical sensor for SARS-CoV-2 detection (Reprinted from Ref. [50]).

Integrating PCR with electrochemical transducers poses various challenges; the primary challenge includes the capability of the sensing surface to withstand the harsh temperature changes and salt concentrations required during PCR [49]. Isothermal amplification techniques are preferred over PCR for integration with electrochemical sensors. A rapid electrochemical detection system based on rolling circle amplification (RCA) was demonstrated for multiplex detection of the S and N genes of SARS-CoV-2 [50] (Figure 3). Sandwich hybridization was employed in this study, with oligonucleotide probes consisting of redox-active labels (methylene blue-and-acridine orange) for electrochemical detection using differential pulse voltammetry. This assay detects the N or S viral gene at a concentration as low as 1 copy/μL within 2 h with high selectivity and sensitivity.

The recent advances in microfluidics technology have enabled the integration of electrochemical electrodes with miniaturized reaction chambers (or chips) designed for PCR. The USFDA recently approved the GenMark ePlex^®^ SARS-CoV-2 test, which automates RNA extraction and amplification, and then further integrates it into competitive hybridization-based electrochemical detection [51]. This system uses the principle of electrowetting (digital microfluidics) to manipulate the movement of samples and reagents on a printed circuit board (PCB) (Table 1).

## 4. CRISPR/Cas-Based Sensors: The New Alternative

CRISPR stands for clustered regularly interspaced short palindromic repeat, which utilizes genetic information of bacterial species as a part of an antiviral process. CRISPR/Cas is a genetic editing technology whose precise and specific DNA and RNA cleavage ability makes it a useful tool in nucleic acid diagnostics. CRISPR/Cas-based sensors mainly utilize single guide RNA in conjunction with the Cas system to bind to a target sequence or cleave target DNA and RNA, resulting in signal generation. Owing to their high specificity, they are an attractive alternative to POC RT-PCR devices. CRISPR/Cas-based diagnostics circumvents the issue of long turnaround times and enhances the assay specificity [52]. Recently, Hou et al. developed a rapid assay known as CRISPR–COVID for detecting SARS-CoV-2 with less turnaround time (~40 min) compared with RT-PCR and metagenomics sequencing [53]. Another advantage of using CRISPR/Cas systems is the exclusion of RNA isolation and amplification, making it a faster analysis method. An ultrasensitive RT-RPA CRISPR–fluorescence detection system (FDS) assay can eliminate the need for RNA isolation for SARS-CoV-2 detection [54]. It uses a saliva sample that is subject to a mix of chemicals that amplify the viral RNA, which is then subjected to CRISPR/Cas12a-based fluorescence signal amplification. The linear range of this handheld CRISPR-based test was found to be 1 to 10^5^ copies/mL with a limit of detection of 0.38 copies/mL, which is consistent with the result obtained using qRT-PCR. In another approach, the need for SARS-CoV-2 RNA pre-amplification was eliminated with the use of CRISPR-Cas13a, which aids the detection of SARS-CoV-2 RNA from nasal swabs [55]. The main highlight of this study was the use of different sets of crRNAs to increase the sensitivity by activation of a greater number of Cas13a per target RNA. Additionally, the study reported the ability to directly translate the fluorescent signal into viral loads, thus resulting in remarkable sensitivity compared with other CRISPR-based assays for COVID detection.

## 5. Future Outlook

COVID-19 diagnostics has evolved significantly since its first appearance. The range and types of diagnostic devices that have emerged in the past year are immensely diverse. Several earlier diagnostic devices and assays were only the subject of academic interest and research but are now commercially available for use. However, since most of the POC devices for COVID-19 detection have been authorized under emergency use, caution should be taken when extrapolating the use of such devices for the diagnosis of other diseases.

Despite the advances, there are limitations associated with RT-PCR POC devices and biosensors concerning sample preparation in ePCR, false negatives and positives, and reagent evaporation in dPCR. Efforts to identify the limitations in current PCR devices for COVID-19 detection can soon help in the design of improved diagnostic devices. Additionally, different detection strategies and platforms can be integrated to develop new, hybrid devices for improved performance. For example, electrokinetic focusing on microfluidic chips was used to automate the process of nucleic acid purification and amplification with a reduction in non-specific amplification [56]. A recent study used isotachophoresis (ITP), an ionic focusing technique, on a microfluidic chip to automate SARS-CoV-2 RNA purification and subsequent detection by CRISPR-based technique within 35 min [57]. This on-chip device uses a smaller volume of reagents (<100 times lower) and automates sample preparation and subsequent detection. Reduction in bubble generation and reagent evaporation in dPCR systems was also demonstrated by creating a vertical polymeric barrier leading to ultrafast PCR amplification [58].

Centrifugal microfluidic platforms (or lab-on-a-disc) for automated sample preparation and subsequent RT-PCR can also be conceived. These devices use different layers of polymeric substrates to integrate multiple steps involving complex fluid flow. These centrifugal systems were shown to improve reaction rates using efficient mixing, thus enabling high sensitivity and reduced hybridization times [59]. Paperfluidic devices that involve the creation of microfluidic channels on paper can also be realized for SARS-CoV-2 RNA detection. Apart from being inexpensive, paperfluidic devices do not require any additional step to render the channels hydrophilic for fluid flow; the intrinsic hydrophilicity of paper allows fluid flow via capillary action, thus eliminating the need for external pumps. This allows their use in resource-limited, POC settings. These devices, much like LFAs, can be batch fabricated at minimal cost and can thus be used in mass screening operations in resource-limited settings. A paper-based assay, FnCas9 editor-linked uniform detection assay (FELUDA), was developed in India, which enables detection of single nucleotide variants [60]. This test uses RT-PCR followed by CRISPR-based detection in a lateral flow format. Similarly, paperfluidic devices that can integrate RNA extraction, amplification, and subsequent detection can be realized [61].

COVID-19 diagnostics has provided new opportunities and advances in the clinical diagnostic sector. It will be interesting to see how these developments affect the overall diagnostics landscape over time.

## 6. Conclusions

Molecular diagnostics has been the cornerstone in controlling the ongoing COVID-19 pandemic. RT-PCR is currently the primary gold standard for COVID-19 diagnosis. Simultaneously, this crisis has brought us to realize the importance of low-cost, sensitive, and high-throughput devices that can be deployed in POC settings. On-site analysis that is fast, reliable, and helps to reduce the economic costs of infection transmission and potential quarantine is required. Different rapid POC tests have been authorized and deployed for mass screening and diagnostic purposes. Yet, RT-PCR has remained the primary and the only method for COVID-19 confirmation. Miniaturized PCR and PCR biosensors, devices that integrate PCR with different detection modalities, have emerged as tools that can address the issue of the low sensitivity of the current rapid POC tests and simultaneous analysis of samples in a high-throughput manner outside of a centralized lab. Digital PCR has emerged as an efficient high-throughput system. However, it does not eliminate the use of expensive reagents and often requires professional involvement in its operation. Electrochemical PCR is also a viable option for faster, cost-effective, and sensitive COVID-19 detection. However, the difficulty of the integration of PCR with electrochemical systems still creates formidable challenges in realizing a commercially adaptable system. CRISPR/Cas-based systems have further created a scope for diagnostic devices that do not require RNA extraction and amplification before detection.

The active transition from routine diagnostic laboratories to the realm of high sensitivity molecular diagnostics can significantly increase the efficiency and responsiveness of POCTs and facilitate the management of outbreaks in difficult settings. Devices such as those mentioned above can readily aid healthcare professionals in making faster medical decisions. However, there are still limitations to be addressed in such systems. Sample preparation errors and false positives and negatives need to be addressed before these assays can eventually be used for other diagnostic applications as well. Although different formats of POC RT-PCR assays have emerged, there is still scope for the development of hybrid, integrated systems that have better performance in terms of specificity and response time. Rigorous validation protocols and a high sampling rate would determine whether these devices are capable of use in the long run.

## Figures and Tables

**Table 1:** List of commercial, automated RT-PCR systems authorized under emergency use.

Name of the Kit	Target Genes	Type	Sample Preparation	No. of Tests	Time	LOD	Sensitivity	Specificity	Cost (Per Test)	Reference
CovidNudge	rdrp1, rdrp2, E gene, N gene, n1, n2, and n3	RT-PCR	Automated	NA	~90 min	5 copies/µL	>94%	100%	GBP 10	[20]
Accula SARS-CoV-2 Test	N gene	RT-PCR	Automated	NA	~30 min	NA	100%	100%	USD 20	[22]
Cepheid Xpert Xpress SARS-CoV-2 assay	N2 and E	RT-PCR (real time)	Automated	10 per kit		0.02 PFU/mL			USD 19.8	[23]
FastPlex Triplex SARS-CoV-2 Detection Kit	ORF1ab, N, RPP30	RT-dPCR	Manual	96 test per kit	90 min	285.7 copies/mL	>95%	95.7%	USD 1152	[23]
Gnomegen COVID-19 RT-Digital PCR Detection Kit	N1, N2	RT-dPCR	Manual	48 samples per day	180 min	2.5 copies per reaction	>95%	99%	NA	[23]
Bio-Rad SARS-CoV-2 ddPCR Test	N1, N2	RT-dPCR	Manual	96 samples	NA	400 copies/mL			NA	[23]
ePlexSARS-CoV-2 Test	N gene	End-point RT-PCR with electrochemical Detection	Automated	12 tests/kit	NA	1 × 10^3^ copies/mL	99.02%	98.41%	NA	[23]

## Data Availability

Not applicable.
